# Modulus of Natural Rubber Cross-Linked by Dicumyl Peroxide III. Some Molecular Interpretations, Possible Refinements of Theory, and Conclusions

**DOI:** 10.6028/jres.080A.051

**Published:** 1976-06-01

**Authors:** Lawrence A. Wood

**Affiliations:** Institute for Materials Research, National Bureau of Standards, Washington, D.C. 20234

**Keywords:** Cross-linking of rubber, dicumyl peroxide, elasticity theory of rubber, entanglements in rubber, gel point, molecular interpretation of rubber elasticity, modulus of rubber, rubber elasticity, statistical theory of rubber elasticity, swelling of rubber network

## Abstract

The shear modulus *G* = 5.925 × 10*^−^*^3^(*fp* − 0.45)*T*+G* (Part I), its energy component G* = 0.0684 (*fp* − 0.45)+ 2.70 (Part II), and the number of effective suh-chains per unit volume *v_e_*= (G – G*)*/RT* are given detailed molecular consideration. *G* is given in Mdyn cm^−2^ for rubber cross-linked by adding *p* parts of dicumyl peroxide per hundred of rubber, and heating until a fraction *f* of the peroxide is decomposed. *v_e_* is found to be approximately twice the density of cross-links, after a correction for impurities and chain ends is made. It can not be computed as *G/RT* since only the entropy component of modulus is related to *v_e_.* The sub-chains for the most highly cross-linked rubbers studied had a molecular weight of about 575 g mol^−1^, corresponding to about 8 isoprene units. The modulus corresponding to no added cross-links is not zero. It is determined chiefiy by the energy component of the modulus; it does not arise from entanglements. The “front factor” is found to be unity.

An extensive literature survey yields values of the quantity *RT*Ψ(*v*_2_), where Ψ (*v*_2_) is the Flory- Rehner equation function of *v*_2_, the equilibrium volume fraction obtained by swelling the cross-linked rubber. *RT*ψ (*v*_2_) is found to be greater than G – G* but not as large as *G* itself.

## 1. Introduction

The first paper in this series Part I [1]^1^ presented experimental data on the change of modulus with temperature and cross-linking for natural rubber cross-linked by dicumyl peroxide.

The results were presented in the form of the following equation:

$$G=S\left(fp+B\right)T+H\left(fp+B\right)+A=5.925\times 10^{-3}\left(fp-0.45)T+0.0684(fp-0.45\right)+2.70$$ (1.1)

*S, B*, *H*, and *A* are constants having the values shown, while *G* is the shear modulus (limit of the ratio of shear stress to strain at zero deformation, in Mdyn cm^−2^ or 0.1 MPa) at a temperature *T* in kelvins, for natural rubber cross-linked by adding *p* parts of dicumyl peroxide per hundred of rubber (phr) and heating until a fraction *f* of the peroxide was decomposed. The actual behavior of the system at very low degrees of cross-linking deviated from that given by the equation, and is discussed in a later section of the present paper.

The second paper Part II [2] applied thermodynamical considerations to the experimental results to determine the relative importance of G* (*= H*(*fp* + *B*) + *A*)—the energy component of the modulus—and (G – G*)—the entropy component. The experimental entropy component was then compared with that predicted by the statistical theory of rubber elasticity in an extremely simplified presentation.

The general concepts outlined in the books of Flory [3], Treloar [4], and Ferry [5] were utilized, largely using the form and symbolism employed in the book by Meares [6].

The present paper makes much more detailed molecular interpretations of the results than the second paper and examines the consequences of several refinements which might be made in the application of the theory.

## 2. Molecular Interpretations

### 2.1. The Gel Point

The gel point or degree of cross-linking just necessary for the formation of a network has been determined experimentally in Parts I and II by noting the degree of cross-linking at which there is no change of modulus with temperature (i.e., *∂G/∂T*= 0). At lower degrees of cross-linking this derivative is negative, as normally observed for uncross-linked polymers [7–9]. At higher degrees it is positive, as is normal for cross- linked polymers.

The gel point is reached only when the cross-linking has attained some characteristic value. In the present study it was found experimentally that 0.45 phr of dicumyl peroxide must be added to attain the proper degree of cross-linking. Some of this represents dicumyl peroxide wasted by reaction with impurities in the rubber, and the remainder is required to produce, on the average, one cross-link per rubber molecule.

Our calculations of the relative sizes of these components have been based on an assumption regarding the amount of impurities in the rubber. However, the total amount of added dicumyl peroxide required in our work is 0.45 phr regardless of the relative sizes of the components and regardless of the value of the constant *H* in eq (1.1)

No attempt was made in Part II in the section dealing with the theory of rubber elasticity to predict a value for G*, the modulus of the rubber at the gel point. The theory was used to predict only G – G*, the increase of modulus caused by the addition of active sub-chains beyond this critical amount of crosslinking.

The modulus of the network at the gel point at all temperatures in the present study is found by direct experiment to be equal to *A* = 2.70 Mdyn cm^−2^ regardless of whether the constant *H* is zero, as in eq (1.2) of Part II; or 0.0684 Mdyn cm^−2^ (phr)^−1^ as in eq (1.1) of Part II (eq (1.1) of this paper).

The statement may be readily verified by an examination of figure 2 of Part II. The modulus at the gel point is likewise independent of the value assumed for the dicumyl peroxide wasted by reaction with impurities, although this is one of the factors determining the abscissa of the gel point.

It is quite surprising that many previous workers have generally neglected consideration of the gel point and its effects on modulus. Most authors, with certain notable exceptions [10–18] have even failed to mention its existence. Charlesby [12] has given one of the most extensive discussions of the subject.

The nonzero value of the modulus near the gel point has likewise not been clearly demonstrated previously, although the experimental evidence has been available.

### 2.2. Alternative Methods of Expressing Added CrossLinking and Related Quantities

In Parts I and II, the amount of added cross-linking has been expressed in terms of the quantity *fp*, since that is the independent variable directly observed experimentally. It is of course pertinent only to the system of natural rubber cross-linked by dicumyl peroxide. For comparison with other systems and other measured properties the cross-linking can be alternatively expressed in terms of other quantities also closely related to A, the number of added cross-links.

Utilizing the numerical values found in Part I for the constants, we shall give the expression for calculating each of these quantities in turn in terms of the quantity *fp.* For convenient reference all the numerical values used in the present paper are summarized in the Appendix. Some of the new expressions have a greater significance in terms of molecular considerations than those using *fp.* However, the values are dependent on the validity of the theory outlined in Part II and on the constants which have been determined from it. Some of the values of course apply only to the particular sample of rubber studied here. Unlike *fp*, the values considered here are not directly observable quantities.

One of the most fundamental of these quantities is *X/N*, the number of added cross-links per rubber molecule. With the insertion of the values of:

*fw* (amount of dicumyl peroxide wasted by reaction with impurities)
*M* (average molecular weight of uncured rubber), and
*M_d_* (molecular weight of dicumyl peroxide) eq (3.7) of Part II becomes

$$X/N=\left(M/100M_{d}\right)\;\left(fp-fw\right)=7.14\;\left(fp-0.31\right).$$ (2.2.1)

Table 1 shows values of *X/N* calculated from this equation for values of *fp* up to 23.8 phr, the highest degree of cross-linking reached in the present study. It will be noted that there is one added cross-link per molecule at the gel point (*fp* = 0.45 phr) as expected. At the highest cross-linking, there are 168 cross-links per molecule on the average.

The cross-linking can also be expressed in terms of the number of moles of unwasted decomposed dicumyl peroxide per unit volume, which has been assumed to be the same as the number of moles of added crosslinks per unit volume. This quantity is found from eq (3.6) of Part II to be

$$X/(A_{1}V_{r})=[1/100M{\;}_{d}{\bar{v}}_{r})][fp-fw]=33.399(fp-0.31)\times 10^{-6}$$ (2.2.2)

where *A*_1_ is Avogadro’s Number, *V_r_* the volume of rubber considered and ${\bar{v}}_{r}$ the specific volume of the rubber.

The values are given in table 1 in units of *μ*mol cm^−3^—numerically equal to the SI units of mol m^−3^.

This quantity may also be weritten as $\left(1/2\right){\bar{v}}_{r}^{-1}M_{c\;chem}^{-1}$ where *M_c chem_* is the molecular weight of a sub-chain (i.e., between cross-links), as determined from stoichiometry. Thus

$$(1/2)M_{c\;chem}^{-1}=(1/100M_{d})\;(fp-fw)=36.986\;(fp-0.31)\times 10^{-6}$$ (2.2.3)

Values calculated from this equation are also shown in table 1.

At the highest cross-linking the molecular weight of an average sub-chain is seen to be 575 g mol^−1^. Each average sub-chain would thus be made up of 8.44 isoprene units and would contain about 34 main-chain carbon atoms.

**FIGURE 1:**
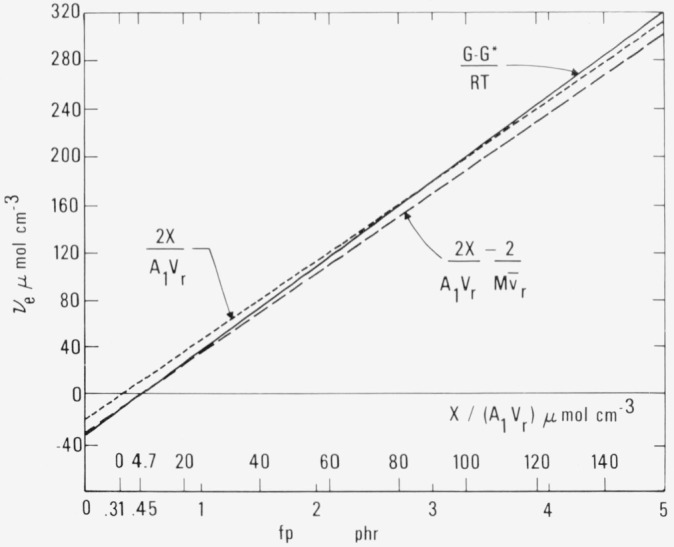
Effective sub-chain density (moles of effective sub-chains per unit volume) as a function of amount of decomposed dicumyl peroxide or as a function of added cross-linking. _______ Continuous line represents eq (2.3.2)………… Dotted line corresponds to 2 sub-chains for each added cross-link, uncorrected for chain ends.---------- Dashed line represents eq (2.3.1). Lower scale of abscissas shows values of fp for system of natural rubber cross-linked by dicumyl peroxide. Upper scale of abscissas shows number of moles of added cross-links per unit volume *X/(A_1_V_r_*) or 1/2*v_r_^−l^M_c_*^−1^*_chem_*, derived from eq (2.2.2).

**FIGURE 2:**
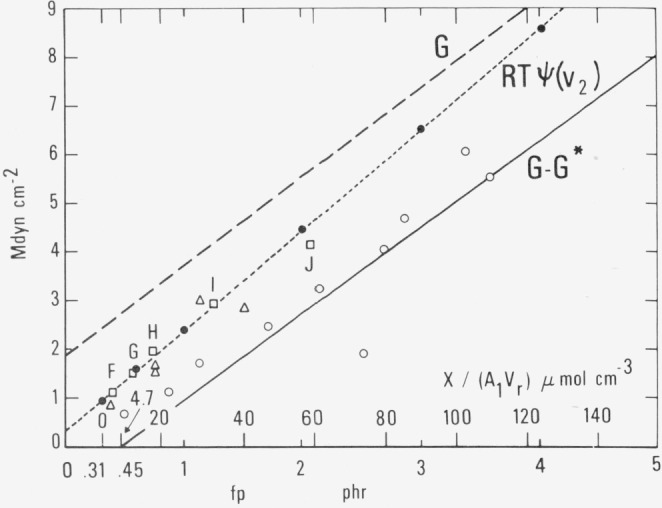
Modulus G at 298.15 K or its entropy component (*G* – G*) from indentation measurements as a function of cross-linking. Comparison with equilibrium swelling results
…………… Dashed line represents the equation *G*= 1.835 (*fp* − 0.45)+2.70, obtained from eq (1.1) at 298.15 K________ Continuous line represents the entropy component G – G*= 1.766 (*fp* − 0.45), obtained from eq (2.3.2) at 298.15 K.…………… Dotted line represents *RT*Ψ (*v*_2_) namely $RT[-\ln(1-v_{2})-v_{2}-\mu v_{2}^{2}][V_{1}{\;}^{-1}]{\left[v_{2}{\;}^{1/3}-v_{2}/2\right]}^{-1}$, right-hand member of Flory-Rehner equation, eq (2.4.1) at 298.15 K with *v*_2_ data from equilibrium swelling measurements of van der Hoff [21]. Empirical equation of line: *RT*Ψ(*v*_2_) = 2.05*fp* +0.347. Data points from equilibrium swelling measurements of:
● van der Hoff [21] (unpurified sample)⊡ Chasset and Thirion [23] or Plazek [29]△ Allen et al. [36].○ Tamura and Murakami [38]. Lower scale of abscissas shows values of fp for system of natural rubber cross-linked by dicumyl peroxide. Upper scale of abscissas shows number of moles of added cross-links per unit volume *X/(A_1_V_r_*) or $1/2{\bar{v}}_{r}{\;}^{-1}M_{c\;chem}^{-1}$ derived from eq (2.2.2).

**FIGURE 3:**
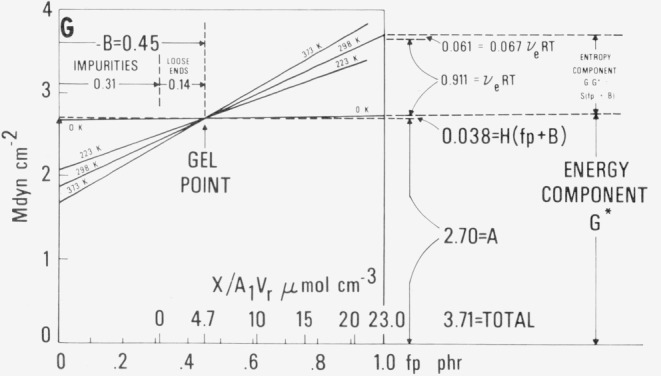
Resolution of modulus *G* (for *T* =298.15 K and *fp* = 1 phr) into components.

For comparison with cross-linking produced by systems other than dicumyl peroxide the quantity *X/*(*A*_1_*V_r_*), the number of moles of added cross-links per unit volume of rubber is preferred. This is shown in figures 1, 2, and 3 as the upper scale of abscissas.

### 2.3. Number of Effective Sub-chains

Aside from the energy component of the modulus, the fundamental quantity governing the physical properties of the cross-linked network is the number of effective sub-chains. This may be expressed in terms of the calculated number of effective sub-chains per molecule, (*2X/N −* 2), given in table 2 and calculated from eq (2.2.1), furnishing the figures given in table 1. However, for many purposes it is most convenient to express this dependent variable as *v_e_* the number of moles of effective sub-chains per unit volume, which may be calculated from *fp* by inserting in eq (3.8) of Part II the values of the constants determined in Part II.

$$\begin{array}{l} v_{e}=[2/{\bar{v}}_{r}][(1/100M_{d})\;(fp-fw)-1/M\\ \;=\left[66.798\;\left(fp-0.31\right)-9.36\right]10^{-6}\\ \;=66.798\;\left(fp-0.45\right)10^{-6}. \end{array}$$ (2.3.1)

From this we conclude that in our rubber there are 9.36 *μ*mol of dangling ineffective loose ends in each cubic centimeter (equivalent to one added crosslink per rubber molecule or 2 sub-chains per molecule) at all degrees of cross-linking. Figure 1 presents a graph (dashed line) of this equation and table 2 shows values calculated from it. It can be seen that the line is displaced from the origin by an amount *fp* =0.45 phr, corresponding to the sum of the shifts, 0.31 phr due to impurities in the rubber and 0.14 phr due to dangling loose ends of rubber molecules.

We find that *v_e_* is zero for *fp* = 0.45 phr, where one cross-link per molecule has been added. Above this point it increases linearly with *fp.*

The dotted line in figure 1 shows the result of omitting completely any correction for the end-effect. It represents the behavior expected if the molecular weight of the polymer had been assumed to be infinite.

For comparison with these values predicted by crosslinking considerations from simple theory, the observed value of *v_e_* may be obtained directly experimentally from the observations of the modulus increase (G – G*). With the insertion of the constants from eqs (1.1) and (2.3) of Part II, eq (3.9) of Part II becomes

$$\begin{array}{l} v_{eG}=\left(G–G*)/RT=\left(S/R\right)\;(fp+B\right)\\ =71.26\;\left(f–0.45\right)\times 10^{-6}. \end{array}$$ (2.3.2)

The subscript *G* has been added temporarily at this point to emphasize that this *v_e_* has been obtained experimentally from the observed values of (*G* – G*) and not from cross-linking considerations. This equation is represented by a continuous line in figure 1. The positive ordinate values are 6.7 percent higher than those given by eq (2.3.1). The discrepancy arises from the 6.7 percent difference in the observed and predicted values of the constant *S* already mentioned in Part II and tentatively ascribed to the effect of entanglements. These lines illustrate graphically the extent of the agreement between the number of effective sub-chains calculated from the observed modulus and that calculated from the added cross-links. With the correction for wasted dicumyl peroxide and for the chain ends, the relation is surprisingly close to demonstrating the production of two effective sub-chains for each cross-link formed over the range shown in figure 1. This was of course assumed in the derivation of *v_e_* in eq (2.3.1) by crosslinking considerations, but this assumption has sometimes been questioned.

The conclusion may also be phrased in slightly different terms. The equation of the continuous line in figure 1 in terms of the upper abscissa is obtained by combining eqs (2.2.2) and (2.3.2) to give

$$v_{eG}=2.134\;(X/A_{1}V_{r})-10\times 10^{-6}.$$ (2.3.3)

Each mole of added cross-links thus leads to 2.134 moles of effective sub-chains. If 6.7 percent of these are ascribed to entanglements it may be concluded that our experiments show that each molecule of decomposed dicumyl peroxide gives rise directly to exactly one new cross-link and two new sub-chains.

While one is justified in calculating *v_e_* by dividing (*G* – G*) (the entropy component of the modulus) by *RT*, as has just been done, no significant information regarding the number of effective sub-chains can be obtained from *G* or G* alone. The ratio of either of these single quantities to *RT* is not a proper measure of any kind of sub-chain density, since the component G* is not related to entropy. Unfortunately most previous authors have based calculations of physically effective sub-chain density on the ratio *G*/(*RT*). This can be valid only if G* is neglible compared with *G*. Table 1 of Part II shows that this is far from true in the present investigation. This point deserves special emphasis because of the confusion in the literature.

It is clear that eqs (2.3.1) and (2.3.2) predict no variation of *v_e_* itself with temperature. However its efficiency in producing an entropy component of the modulus is proportional to the temperature, falling to zero at O K, as is obvious from eq (2.3.2). The entropy component of modulus at a given temperature then bears a close relation to the number of moles of effective sub-chains per unit volume and is in fact equal to *RT* times this number. G*, on the other hand, is an energy component related to intramolecular or intermolecular forces. It is assumed that its magnitude does not vary with temperature, and its variation with crosslinking is found to be very small, arising only from the presence of the term involving the constant *H* in eq (1.1).

A quantity $M_{c\;phys}^{-1}={\bar{v}}_{r}v_{e}$ has also been used by some previous workers to express the concentration of effective sub-chains. From eqs (3.2) and (3.6) of Part II and (2.2.3) of this paper one finds

$$\begin{array}{l} M_{c\;phys}^{-1}=M_{c\;chem}^{-1}-2M^{-1}\\ \;=2{\left(100M_{d}\right)}^{-1}(fp-fw)-2M^{-1}\\ \;=[73.972(fp-0.31)-10.36]10^{-6}\\ \;=73.972(fp-0.45)10^{-6}. \end{array}$$ (2.3.4)

Table 2 shows values calculated by this equation. Similarly one may utilize *v_eG_* instead of *v_e_* to calculate corresponding values for a molecular weight between cross-links as calculated from (G – G*).

In most of our work we prefer to follow the practice shown in figure 1, where the dependent variable is *v_e_*, the number of moles of effective sub-chains per unit volume, and where the independent variable is the cross-linking expressed in terms of *fp* for the dicumyl peroxide system or in terms of *X/A*_1_*V_r_* in general. In this way one avoids the reciprocal quantities and possible confusion involved in using *M_c phys_* and *M_c chem_.*

A comparison of the respective values of *M_c_*_chem_ in the last column of table 1 with those of *M_c_*_phys_ in the last column of table 2 shows the necessity for making a careful distinction between these two quantities. The distinction is always important conceptually and is quantitatively most significant at low degrees of crosslinking. The distinction would disappear for a polymer of infinite molecular weight as can be noted from eq (2.3.4).

### 2.4. Determination of Modulus Components by Measurements of Equilibrium Swelling

As an alternative to experiments involving deformation of cross-linked polymers by mechanical stress, many other investigators, [10–16, 19–39] have measured the swelling of the system by a liquid. The internal stresses arising from the swelling are balanced by the reaction of the extended network. This is observable as an increase of volume, approaching a limiting equilibrium value.

Flory and Rehner [40–42] have utilized statistical considerations to derive a relation intended to permit the calculation of the apparent number of effective subchains from the observed limiting equilibrium swelling. Alternatively this number can be expressed as *v_e_*, ${\bar{v}}_{r}{\;}^{-1}M_{c}-1_{phys}$ or (G – G*)/*RT* as discussed in section 2.3 and plotted in figure 1.

For reasons that will soon become evident, the dependent variable in this section will first be taken, not as *v*_e_, but as (G – G*), the entropy component of the modulus at a temperature of 298.15 K. This variable must be divided by the factor *RT* to obtain *v_e_* itself.

In terms of modulus components, then, the Flory- Rehner equation can be written:

$$\begin{array}{c} G-G*=v_{e}RT={\bar{v}}_{r}{\;}^{-1}M_{c\;phys}^{-1}RT\\ =RT[-\ln(1-v_{2})-v_{2}-\mu v_{2}{\;}^{2}][V_{1}{\;}^{-1}]{\left[v_{2}{\;}^{1/3}-v_{2}/2\right]}^{-1} \end{array}$$ (2.4.1)

where *v_2_* is the measured volume-fraction of rubber in the swollen material, *V*_1_ is the molar volume of the swelling liquid, and *μ* is the Flory-Huggins interaction parameter characteristic of the rubber and swelling liquid. It is normally about 0.4. We shall denote the right-hand member of the equation as *RT*Ψ(*v*_2_) for brevity.

Values of *RT*Ψ(*v*_2_), the right-hand member of eq (2.4.1), calculated from equilibrium swelling data reported by five different observers are plotted as experimental points in figure 2. The results of van der Hoff [21], Chasset and Thirion [23], Plazek [29], and Allen and co-workers [36] are reasonably well represented by the dotted line, which has been drawn to represent them. Similar results (not plotted) were also obtained by Angerer [37]. The results of Tamura and Murakami [38] are consistently somewhat below the dotted line and show a somewhat smaller change with increase of cross-linking.

The data plotted in figure 2 are also typical of the results obtained by many other observers [13–16, 19, 20, 22, 24–28, 30–35]. The latter results are not plotted here in order to avoid complexity. However, the points are generally found to lie near or below the dotted line. In no case do they fall below the plotted results of Tamura and Murakami [38], which are reproduced here to typify the extreme case. Most of the differences among these results can probably be explained in terms of differences in the molecular weight of the rubber, its impurities, or the conditions of cure and swelling.

The continuous line in figure 2 represents a plot of (*G –* G*) at 298.15 K, in accordance with eq (2.3.2). It represents the entropy component obtained in part I of the present study, and differs from the continuous line in figure 1 only by the constant factor *RT* in the scale of ordinates. As already demonstrated in figure 1 the values of *v_e_* calculated from cross-linking considerations differ only slightly from those corresponding to this continuous line. The negative intercept can be ascribed to the presence of chain ends and impurities in the rubber, while the slightly greater slope can be ascribed to the presence of entanglements.

It is clear from figure 2 that the values of *RTΨ*(*v*_2_), the right-hand member of eq (2.4.1), obtained from swelling measurements are systematically substantially larger than the values of (G – G*). In other words we find that the entropy component of the modulus effective in limiting swelling is larger than the entropy component measured by the other methods.

The conclusion then is that the number of sub-chains limiting swelling is larger than the number calculated from cross-linking considerations or its approximate equivalent number effective in the mechanical measurements of the present work.

Furthermore the slope of the dotted line in figure 2 representing the results of the five investigations [21, 23, 29, 36, 37] is about 16 percent greater than that of the continuous line. The comparable figures obtained from the observations of Meissner [14], Manik and Banerjee [31], Mullins [20], and Redding and Smith [32] are 10, 7, 6 and 3 percent respectively. The data of Mason [25] and Tamura and Murakami [38] show even lower slopes, nearly the same as that of the continuous line. Added cross-links are apparently slightly more effective in increasing the entropy component of the swelling modulus than they are in increasing the corresponding entropy component of the mechanical modulus at a given temperature.

These two discrepancies of ordinate values and slopes of the lines in figure 2 are not due to differences in specimens, differences in calculating abscissa values, or difference in the methods used in measuring the modulus by mechanical means, since values of *G* calculated from stress relaxation observations of Chasset and Thirion [23], from creep observations of Dickie and Ferry [30] or from torsion pendulum measurements of Plazek [29] on specimens representing the same samples as those used in their swelling experiments are in excellent agreement with our values. This comparison is shown in figure 8 of Part I. Additional experimental points in good agreement with this figure have been obtained more recently by Gent and Kuan [43] in torsional experiments and by Tamura and Murakami [38] by linear extension.

For comparative purposes figure 2 shows also as ordinate a plot (dashed line) of *G*, the sum of the energy and entropy components of the mechanical modulus at 298.15 K, as obtained earlier by indentation measurements. This, of course, corresponds to eq (1.1) and to one of the lines in figure 2 of Part II. Its slope is about 3.8 percent greater than that of the plot of (G – G*) because of the presence of the term containing the constant *H.* No points in figure 2 and (with scarcely any exceptions) none of the unplotted observations of equilibrium swelling previously mentioned lie above the dashed line. In other words *RTψ(v*_2_) over the range shown never exceeds the total modulus obtained by mechanical measurements. It should be noted that there is no justification for drawing a line in figure 1 to correspond to the line *G* in figure 2, since the energy component of *G* does not arise from any definite number of cross-links, as emphasized in section 2.3.

This same discrepancy of ordinates has already been pointed out by other authors. Dickie and Ferry [30] present a graph (their fig. 2) showing that the equilibrium compliance observed in their creep measurements was smaller by a factor of 0.71 than that calculated by eq (2.4.1) from Chasset and Thirion’s swelling measurements [23]. In terms of the quantities we have used, this means that the directly-observed modulus *G* was found to be greater then *RTψ*(*v*_2_) by about the same factor as we note in the region where *fp* is between 1 and 2 phr. Similar results for natural rubber and polybutadiene in this range were reported by Shen, Chen, Gebhard, and Cirlin [35] and for styrene-butadiene rubber and butyl rubber by Nielsen [44].

Murakami and Hsiue [39] extended the observations on natural rubber to higher degrees of cross-linking than the other observers. They confirmed previous work at low values, but in addition they found that Ψ(*v*_2_) from swelling measurements (which they denoted as *n_s_*) was less than *G/RT* (which they denoted as *n_M_*) only as long as the value of *G/RT* was less than 420 *μ*mol cm^−3^ corresponding to a G value of 10.4 Mdyn cm^−2^ at 298.15 K. Beyond this point the reverse was true.

The relative slopes of the dotted and dashed lines in figure 2 would also indicate the possibility of such a reversal if extrapolation of the lines is justified. The intersection of the lines occurs at an abscissa where *fp* = 7.08 phr (*X/A*_1_ *V_r_* = 226 *μ*mol cm^−3^) corresponding to an ordinate of 14.9 Mdyn cm^−2^ This is not greatly different from the value 10.4 Mdyn cm^−2^ found by Murakami and Hsiue.

In contrast with the precent paper, most previous publications have neglected to include G* in the Flory-Rehner equation and thus have considered its left-hand member to be simply *G*, the sum of the entropy and energy components. If this should be correct, *RT*Ψ(*v*_2_) ought to be compared with *G* rather than with *G*–G*, and it would not be necessary to conclude that there were more cross-links effective in limiting swelling than those calculated by the other methods. From figure 2 one would then conclude that *RT*Ψ(*v*_2_) could now be taken as a total modulus including an energy component which is roughly only about half that effective in the mechanical measurements. If it is assumed that in the swollen system inter-chain forces are very greatly reduced, while the intra-chain forces are not much affected, a tentative guess could be made that these types of forces would be approximately equal in the unswollen rubber. On the other hand, most previous workers consider that the intra-chain forces are strongly predominant. However, the present work yields no information about these forces if the left-hand member of eq (2.4.1) is taken as (*G*–G*), as we have done.

## 3. Possible Refinements of Simple Theory

### 3.1. Impurities and Sol Content

Specific impurities in the rubber which would react with the dicumyl peroxide rendering some of it unavailable for cross-linking have been discussed in sections 3.2 and 4 of Part II. In the absence of direct measurements, the equivalent amount of these impurities in pale crepe rubber was taken as 0.31 phr, as found in swelling measurements made by van der Hoff [21]. Similar measurements on samples of several extracted and unextracted natural rubbers by Bristow, Moore, and Russell [28] and other measurements reported by Bristow [27] yielded values ranging from 0.2 to 0.45 phr, in approximate agreement with van der Hoff’s figure. This number would be expected to vary with the type of rubber studied.

Degradation of the rubber is much more likely with extracted samples, since extraction removes the natural antioxidants. Consequently in most instance preference should be given to results obtained with unextracted samples, making the necessary corrections.

In general, all impurities in the rubber, both reactive and nonreactive, would also act as diluents, for which an additional correction might be made. A similar correction might be made for impurities in the dicumyl peroxide itself. In each case the mass should be multiplied by the corresponding purity.

The purity of the pale crepe rubber may be estimated as normally about 93 percent. The purity of the “recrystallized” dicumyl peroxide was not measured here, but values near 95 percent have been reported [14, 31, 37, 45–48].

The relations derived here are expressed in terms of the ratios

$$p=100m_{d}/m_{r}\;[\text{eq}\;(3.3)\;\text{of Part II}]$$

and

$$w=100m_{o}/m_{r}\left[\text{eq}\;\left(3.4\right)\;\text{of Part II}\right].$$

Therefore the correction factor would involve only the ratio of the purities. It would be unity if the purities of the rubber and dicumyl peroxide should be the same. Since these purities probably were nearly the same in the present work, no correction for diluent effect was made.

The reaction products of the decomposition of the dicumyl peroxide are *α-α*′-dimethyl benzyl alcohol, acetophenone, and methane. It is expected that a portion of the first two products will remain as a residue in the rubber. No significant anomalies due to the presence of these residues were noted in the experimental portion of the present study, and no account was taken of them in the simple theory outlined here. However, a more detailed investigation of the most highly cross-linked specimens might be warranted in order to determine quantitatively the fate of these reaction products and their influence on the properties of the rubber.

The sol content of the cross-linked rubber consists of molecules which never become a part of the network. K. W. Scott [47] measured a sol content of about 1 percent for natural rubber cross-linked with 1 phr of dicumyl peroxide and about 0.1 percent when the amount was 3 phr. Similar results were reported by Glaser and Eirich [33]. Still smaller amounts were found when larger amounts of dicumyl peroxide were used. Consequently the sol content was assumed to be negligible in all the present work.

### 3.2. Specific Volume of Rubber

The specific volume of the unvulcanized rubber (NBS Standard Reference Material 385b) at 298.15 K was 1.1074 cm^3^ g^−1^. For simplicity this value has been used for ${\bar{v}}_{r}$ throughout the present study. The actual specific volume of the cross-linked rubber would be expected to be less than this value because of the diluent effect of the dicumyl peroxide and the change of volume on cross-linking. A decrease of about 10 percent would be expected at the highest degrees of crosslinking. The specific volume would also be expected to vary about 5 percent above and below the value at 298.15 K at the extremes of temperature used in the present study.

### 3.3. Chain-End Correction

The correction for dangling chain ends ineffective for supporting a stress is discussed in Section 3.1 of Part II [2]. The form of correction used there is exactly the same as that proposed by Flory [49]. He used the quantity $M_{c\;chem}^{-1}$ as a measure of cross-linking and so one finds by the use of eq. (2.2.3) in eq (2.3.1)

$$v_{e}=M_{c}^{-1}chem/{\bar{v}}_{r}[1-2/MM_{c}^{-1}{\;}_{chem})].$$ (3.3.1)

Differentiation of this equation gives

$$\partial v_{e}/\partial M^{-1}=-2/{\bar{v}}_{r}.$$ (3.3.2)

In terms of the entropy component of modulus this becomes

$$\partial (G-G*)/\partial M^{{\;}^{-1}}=-2RT/{\bar{v}}_{r}.$$ (3.3.3)

Later workers [50, 51] have proposed slightly different forms of the second term in brackets in eq (3.3.1) [52], while Mullins [20] gives experimental evidence that the factor should be

$$[1-2.3/(MM_{c}^{-1}{\;}_{chem})].$$

It is likely that the factor in this form takes account of the effects of nonload-bearing loops, hitherto neglected, as well as chain ends. Use of the revised factor would increase the calculated value of effective molecular weight by about 15 percent. It would still be in the range of reasonable values. Other refinements also give only small variations in calculated molecular weight.

In figure 1 the whole chain-end correction can be seen to result in shifting the line to the right along the abscissa axis by only 0.14 phr, equivalent to a decrease of 9.36 *μ*mol cm^−3^ on the ordinate axis. This shift is so small that determining the validity of the exact form of correction would require data of high precision extending over a wide range of molecular weights. It would seem that more attention has been given to this correction than would normally be justified by its magnitude. The dotted line in figure 1 is drawn neglecting the correction completely, thus corresponding to an assumption that the polymer was of infinite initial molecular weight. The calculation for a finite molecular weight is of course greatly influenced by the correction for impurities, which in our work was more than twice that ascribed to chain ends.

### 3.4. Modulus at Low Degrees of Cross-Linking

In the experimental portion of the present study (Part I [1]), the conclusions have been based on measurements where *fp* was greater than 0.45 phr, namely beyond the gel point. When *v_e_* is plotted against *fp*, as in figure 1, the considerations already outlined yield the straight lines shown. The value of *fp* at which *v_e_* is zero corresponds to the amount of cross-linking agent which must be introduced to account for that required to establish a network. Only after this amount has been added can any additional cross-linking agent become effective.

At the gel point the number of moles of effective sub-chains in zero (fig. 1) and the modulus *G* is equal to G*, the energy component alone, so that G – G* = 0, as seen in figure 2.

In the remainder of this section we shall consider for the first time the actual observed behavior of the system at low degrees of cross-linking. Below the gel point the network theory outlined previously can make no predictions and we have been guided only by extrapolation. In this region experimental values are less reliable, since the creep rate becomes high [53, 54], and the “equilibrium” modulus is obtainable only by extrapolation to infinite time, for example by the method of Chasset and Thirion [23, 55]. The modulus-temperature relation here is found experimentally to have a negative slope, as predicted by extrapolation from high degrees of cross-linking. This is evident in figures 2 and 7 of Part I.

Results reported by other observers [13, 15–20, 32–34, 36, 48, 56–65] agree with the present work in showing that the modulus increases linearly with crosslinking for the higher degrees of cross-linking. The line likewise almost invariably has a slope greater than that predicted from the cross-linking. In the present work this slope was about 10.5 percent greater than predicted [2], while the literature values in the references just given range from 5 to 31 percent greater.

The line invariably has a positive intercept, ranging from 0.5 to 2 Mdyn cm^−2^. This is in accordance with figure 8 of Part I, figure 2 of Part II, and figure 2 of the present work. Our work shows a value of *Go* = 1.87 Mdyn cm^−2^ at 298.15 K.

The positive intercept and increased slope of the line are evident even when the carbon-carbon crosslinks are formed by exposure to radiation [17, 18, 66] rather than by free radicals resulting from the decomposition of a peroxide. Similar effects are evident in studies of silicone rubber cross-linked by radiation [57, 58].

At the lower degrees of cross-linking the actual measured values of modulus lie consistently below the line, the deviation increasing as the cross-linking is reduced. This behavior can be seen in the modulus of Chasset and Thirion’s Specimen F, plotted in figure 8 of Part I. It is well verified by other observers [13, 15, 17–20, 31, 32, 56, 57, 60, 61, 64]. The experimental values of modulus often appear to decline to zero at a finite positive value of cross-linking, and sometimes are even calculated as a negative modulus for the material to which no cross-links have been added [13, 32].

An examination of the data suggests that as the cross-linking is reduced, the value at which the deviation from linearity first occurs may be the gel point. However, it is possible that the gel point is that at which the modulus actually becomes zero. The experimental and theoretical difficulties associated with consideration of the region near the gel point and below have already been mentioned.

It is interesting to compare our time-independent modulus value of *G*_0_=1.87 Mdym cm^−2^ at 298.15 K, as measured on cross-linked specimens and extrapolated to zero cross-linking, with those derived from observations of a time-dependent modulus obtained from stress relaxation or shear creep experiments on rubber actually containing no cross-linking agent. The latter values have usually been obtained from a point of inflection in the plateau region of a plot of the logarithm of the ratio of stress to strain (or its reciprocal) against the logarithm of the time. Shear creep studies on narrow-distribution synthetic *cis*- isoprene polymers by Nemoto and co-workers [68] yielded shear modulus values of about 0.8 Mdyn cm^−2^ with a plateau centered near −30 °C (243 K). Stress relaxation studies by investigators in the Polytechnic Institute of Milan on various polyisoprenes [69, 70] showed modulus values of 3–4 Mdyn cm^−2^. Such nonzero values of modulus when no cross-links have been added have usually been ascribed to entanglements, which will be discussed in section 3.5.

### 3.5. Entanglements

The linear relation between modulus and crosslinking discussed in the preceding section has a positive intercept Go, the extrapolated modulus corresponding to no added cross-linking agent. *G*_0_ has often been ascribed [19–21, 24, 31, 57, 63, 67, 68] simply to entanglements functioning as effective cross-links, additional to but not much different in character from those which have been introduced by the cross-linking agent. It would seem that this simple explanation must be abandoned in view of the present work, which has now shown that the modulus has a very appreciable energy component G* and that this is the chief factor determining *G*_0_.

The relation between these quantities is given by eq (2.5) of Part II

$$\begin{array}{l} G_{0}=G*+SBT=A+BH+SBT\\ \;=2.67-2.666\times 10^{-3}T. \end{array}$$ (3.5.1)

The values of the intercept *G*_0_ calculated from this equation range from 2.67 Mdyn cm^−2^ at 0 K to 2.07 Mdyn cm^−2^ at 223.15 K and 1.67 Mdyn cm^−2^ at 373.15 K. They can be noted in figure 2 of Part II.

We are led to conclude that *G*_0_ arises from interatomic forces. The consensus of opinion [71–75] has been that these forces are largely intrachain (“intramolecular”) in origin.

The data indicate that these forces (whatever their origin) increase slightly with increasing crosslinking, as might he expected. If entanglements serve to increase these forces, they may well have an important part in giving rise to the intercept.

On the other hand if entanglements act merely as pseudo effective cross-links, they can contribute only to the entropy component of the modulus. In experimental terms, they can increase the slopes of the *G*, *T* lines of figure 1 of Part II but would not affect the intercepts of these lines.

Previous observations of the relation between modulus and cross-linking, with a few exceptions [29, 76, 77] have been limited to measurements at a single temperature or over a small range. Consequently they have not furnished information regarding the relative contributions of energy and entropy.

The theory of entanglements has been extensively discussed in recent years [78–87]. The considerations have now progressed considerably beyond the simple idea of pseudo cross-links just mentioned. For example topological entanglements have been distinguished from coupling loci arising from interatomic forces, and some entanglements are regarded as trapped between cross-links while others are untrapped. Furthermore entanglements are important in determining the viscoelastic and other properties of a given polymer. We restrict ouselves here to considering their effect on the modulus.

Some theoretical treatments predict that the number of entanglements should increase linearly with an increase in the number of cross-links, while others conclude that it should be independent of the crosslinking. The present work would favor the former conclusion, in view of the fact that the observed value of the constant *S* in eq (1.1) is greater than that computed from the molecular constants in Part II. If this increase is indeed due to entaglements and not to some defect in the assumptions, one would conclude that the number of entanglements is consistently about 6.7 percent of the number of cross-links. We know of no independent method by which to verify this conclusion.

If entanglements are regarded as temporary crosslinks [30, 67, 88] which are effective only for a limited time after the application of a stress, they need be considered only when there is appreciable creep. The significant portions of the present work were done under conditions where the creep was negligible. Consequently, here we can neglect the effect of such temporary cross-links.

The scope of the present paper does not permit further discussion of entanglements, other than to repeat that they are not functioning as pseudo crosslinks making a major contribution to the value of Go.

### 3.6. “Front Factor” and Efficiency

One of the most significant conclusions of the present work is the prediction of the value of the constant *S* in eq (1.1) as equal to 2 $R/(100M_{d}{\bar{v}}_{r})$, by eq 4.3 of Part II.

Regardless of whether the 6.7 percent excess of the measured over the calculated value is ascribed to defective assumptions or to the presence of entanglements, as has been done in a previous section, it is clear that the extent of the numerical agreement apparently eliminates the necessity for several refinements and corrections which might he made in the simple theory outlined here.

There has been considerable discussion regarding a “front factor” *ϕ*, [89–91] which might be used in calculating modulus change from *v_e_* the number of added effective sub-chains per unit volume. James and Guth [10, 91, 92] associate *ϕ* with the details of the reaction producing cross-links and suggest a value of about 0.5 as most reasonable. Others [93] have criticized this treatment and predict a value near unity.

Most recent authors have included a different “front factor” $\langle {\bar{r}}_{i}^{2}\rangle /\langle {\bar{r}}_{0}^{2}\rangle$ where $\langle {\bar{r}}_{i}^{2}\rangle$ is the mean square value of the displacement length of the sub-chain in the isotropic unstrained state and $\langle {\bar{r}}_{0}^{2}\rangle$ is the mean-square value of the displacement length in the unperturbed state [74]. The latter quantity varies with temperature.

The assumption that each molecule of decomposed dicumyl peroxide gives rise to one cross-link in natural rubber is generally accepted [21, 31, 94–97] but the efficiency ϵ has occasionally been stated to be less than unity under certain circumstances [27, 28, 60], (especially if no account is taken of the amount wasted by reaction with impurities). Scission during cure would also reduce the efficiency, but has recently been shown to be negligible [28].

The introduction of a “front-factor,” whatever its origin, and an efficiency factor e into eq (3.9) of Part II or eq (2.3.2) of the present paper would result in the substitution of *ϕϵR* for *R*, the gas constant, in all the relations containing *R.* The present results indicate that the product *ϕϵ*, even without the entanglement correction, could not differ from unity by more than about 7 percent. A significant variation of this quantity with temperature would have been observable as a deviation from linearity in the graph of *∂G/∂fp* against *T* and of *∂G/∂T* against *fp* (figs. 6 and 7 respectively of Part I).

If the front factor ϕ is taken as 0.5 the efficiency would have to be an unlikely 200 percent (i.e., each molecule of decomposed peroxide would have to furnish two cross-links yielding four additional sub-chains). Such a counterbalance of factors does not seem probable and we prefer to consider that the present results indicate values of unity for both ϕ and ϵ under our conditions of cure.

## 4. Resolution of Components of Modulus

Many of the concepts developed here can be more readily understood when they are utilized to resolve the modulus at *fp*=l phr, and *T* = 298.15 K, into four components, as shown in figure 3. The first and largest component, corresponding to the constant *A* in eq (1.1), is an energy component arising from interatomic or intermolecular forces. It is represented by a vertical displacement of 2.70 Mdyn cm^−2^ from the origin. The amount of dicumyl peroxide wasted by reaction with impurities is shown as a horizontal displacement of 0.31 phr, while the effect of dangling loose ends of the rubber molecules is given by an additional horizontal displacement of 0.14 phr to the gel point. The second component, also an energy term independent of temperature, corresponds to the term containing the constant *H* and is the smallest of the four components. The third term, an entropy term proportional to the temperature, is the one which can be calculated as *RT* times the density of effective sub-chains attributable to the added dicumyl peroxide. Finally, there is a fourth term, which is also an entropy term proportional to the temperature and the cross-linking. This term can probably be ascribed to the effect of entanglements, functioning as pseudo cross-links.

The lines shown in figure 3 are to be regarded as schematic, since they are the extrapolations of lines determined largely at higher values of cross-linking. In the present work (except in sec. 3.4), little significance has been attached to actual experimental values in the region of the gel point and below.

In view of the fact that a large portion of the modulus arises from an energy component G* (especially at low degrees of cross-linking), it is not surprising that the form of stress-strain relation derived from entropy considerations alone gives only an approximation to the experimental data [63, 73, 90], the value predicted being consistently higher than the observed stress [98, 99]. It will be noted that the present work has required no assumptions whatever about the equation of state or form of the stress-strain relation outside the range of infinitesimal deformations.

## 5. Conclusions

The qualitative and quantitative agreement of predictions and results demonstrated in the previous sections is a strong confirmation of the essential validity of all the extremely simplified molecular considerations involved, including the general aspects of the statistical theory of rubber elasticity. We know of no previous experimental study extending over as wide ranges of cross-linking and temperature. In fact the cross-linking and temperature have been varied simultaneously on only a few occasions in previous work.

An important advantage of the present work over many previous studies is the fact that measurements are made at very small deformations. Thus the results are expressed in terms of the modulus, defined as the limiting value of the ratio of stress to strain at zero deformation. Consequently, the results are independent of the stress-strain relation or equation of state. This means that no consideration needs to be given here, for example to the difference between the stress-strain relation predicted by the statistical theory of rubber elasticity and that given by the Mooney-Rivlin equation or the empirical equation of Martin, Roth, and Stiehler [99].

The present study has shown that the modulus G includes a considerable component arising from internal energy changes as well as that arising from entropy changes. The energy component at room temperature is of the order of half the total when the degree of crosslinking is that normally used with dicumyl peroxide rubbers.

It is concluded that the nonzero value of the modulus when extrapolated to zero cross-linking is due to the energy component of the modulus rather than to entanglements. Entanglements acting as pseudo-cross-links would serve to increase only the entropy component.

The gel point, defined as the minimum degree of cross-linking required to form a network, may be located experimentally as the cross-linking at which the slope of the modulus-temperature relation is zero. The value of the modulus *G* at the gel point is not zero, but is the energy component under this condition; the entropy component of *G* at the gel point is zero.

The amount of dicumyl peroxide required to crosslink rubber to the gel point is the sum of that wasted by reaction with impurities in the rubber and that required to give one cross-link for each rubber molecule. The former quantity was about twice the latter in the work reported here.

The entropy component of the modulus as determined from reported values of equilibrium swelling by the Flory-Rehner equation, is found to be significantly larger than that determined from mechanical measurements. However, the quantity computed is smaller than the sum of the entropy and energy components as determined from cross-linking considerations or from mechanical measurements. It increases linearly with increase of cross-linking at a slightly greater rate than the modulus or the entropy component of the modulus.

It is concluded that the “front factor” sometimes introduced in statistical theory considerations cannot differ from unity by more than about 7 percent. The difference is even less than this if allowance is made for entanglements functioning as pseudo-cross-links.

## Figures and Tables

**TABLE 1:** Calculated measures of added cross-linking

*fp* phr	*X/N* added crosslinks per molecule	*X/(A*_1_,*V*_*r*_) *μ*mol added cross-links per cm^3^	(1/2) *M_c_*^−1^*_chem_* *μ*mol g^−1^	*M_c chem_* mol wt between added cross-links g mol^−1^

0	…………	…………	…………	…………
.31	0	0	0	∞
.45	1.00	4.68	5.18	96,500
1	4.93	23.0	25.5	19,610
2	12.1	56.4	62.5	8,000
3	19.2	89.8	99.5	5,030
4	26.3	123	136	3,680
5	33.5	157	173	2,890
10	69.2	324	358	1,400
15	105	491	543	921
20	141	658	728	687
23.8	168	784	869	575

**TABLE 2:** Calculated measures of effective sub-chains

*fp* Phr	(2*X/N) –* 2 effective sub-chains per molecule	*v_e_ μ*moles of effective sub-chains per cm^3^	$M_{c\;phys}^{-1}\;\mu \;{\text{mol g}}^{-1}$	*M_c phys_* mol wt of effective sub-chains, g mol^−1^

0	…………	…………	…………	…………
0.31	…………	…………	…………	…………
0.45	0	0	0	∞
1	7.86	36.7	40.7	24,600
2	22.2	104	115	8,700
3	36.4	170	189	5,290
4	50.6	237	263	3,800
5	65.0	304	337	2,970
10	136	638	706	1,420
15	208	972	1080	926
20	280	1306	1450	690
23.8	334	1560	1730	578

## Footnotes

1 Figures in brackets indicate the literature references at the end of this paper.

## 6. Appendix

### Numerical Values of Constants

For convenient reference all the numerical values used in calculations in the present paper are given here.

#### GIVEN VALUES

From Tables

Molecular Weight of dicumyl peroxide *= M_d_* = 270.37 g mol^−1^ Gas Constant = *R* = 8.31441 × 10^7^ erg mol^−1^ K^−1^

Observed Values –Part I

${\bar{v}}_{r}=1.1074\;{\text{cm}}^{3}\;g^{-1}$; ${\bar{v}}_{r}{\;}^{-1}=0.90302\;g\;{\text{cm}}^{-3}$
100*M_d_*/*M* = − *B* −*fw* = 0.45 − 0.31 = 0.14
*S* = 5.925 × 10^−3^ Mdyn cm^−2^ K^−4^ phr^−1^
*A* = 2.70 Mdyn cm^−2^
*H* = 0.0684 Mdyn cm^−2^ phr^−1^

**Table:**

VALUES CALCULATED FROM GIVEN VALUES		
		Used in eq
(100*M_d_*)^−1^	= 36.986 *μ*mol g^−1^	2.2.3
2(100M*_d_*) ^−1^	= 73.972 *μ*mol g^−1^	2.3.4
${\bar{v}}^{-1}{\left(100M_{d}\right)}^{-1}$	= 33.399 *μ*mol cm^−3^	2.2.2
$2{\bar{v}}_{r}{\;}^{-1}{\left(100M_{d}\right)}^{-1}$	= 66.798 *μ*mol cm^−3^	2.3.1
$2R{\bar{v}}_{r}{\;}^{-1}{\left(100M_{d}\right)}^{-1}$	= 5.5539 × 10^−3^ Mdyn cm^−2^ K^−1^ phr^−1^	4.3. of Part II
0.14(100*M_d_*)^−1^ = *M^−^*^1^	= 5.18 *μ*mol g^−1^	…………
*2 M*^−1^	= 10.36 *μ*mol g^−1^	2.3.4
$2{\bar{v}}_{r}{\;}^{-1}M^{-1}$	= 9.36 *μ*mol cm^−3^	2.3.1
*M*	= 193,000 g mol^−1^	…………
*M*(*100M_d_*)^−1^	= 7.14	2.2.1
*S/R*	= 71.26 *μ*mol cm^−3^ phr^−1^	2.3.2
